# Sex-Related Differences in Hematological Parameters and Organosomatic Indices of* Oreochromis niloticus* Exposed to Aflatoxin B_1_ Diet

**DOI:** 10.1155/2017/4268926

**Published:** 2017-10-02

**Authors:** Esther Marijani, Johnson Nasimolo, Emmanuel Kigadye, Gbemenou Joselin Benoit Gnonlonfin, Sheila Okoth

**Affiliations:** ^1^The Open University of Tanzania, P.O. Box 23409, Dar es Salaam, Tanzania; ^2^School of Biological Science, University of Nairobi, P.O. Box 30197, Nairobi 00100, Kenya; ^3^Department of Veterinary Anatomy and Physiology, University of Nairobi, P.O. Box 30197, Nairobi 00100, Kenya; ^4^Department of Biology, Catholic University of Eastern Africa, P.O. Box 62157, Nairobi 00200, Kenya

## Abstract

A 24-week feeding experiment was conducted to assess whether males and females of* Oreochromis niloticus* exhibit differences in their hematological responses and organosomatic indices to dietary AFB_1_ contamination. Triplicate groups of* O. niloticus* (initial body weight: 24.1 ± 0.6 g) were fed with four diets (Diets 1 to 4) containing 0, 20, 200, and 2,000 *μ*g AFB_1_ kg^−1^. A significant decrease (*P* < 0.05) in hemoglobin (Hb), red blood cells (RBC), and hematocrit (Hct) was observed in AFB_1_ exposure groups, with the lowest levels recorded in the 2000 *μ*g AFB_1_ kg^−1^ treatment. A significant increase in mean white blood cells (WBC), neutrophils, and lymphocytes was observed in AFB_1_ exposure groups. No sex-related differences in RBC, WBC, lymphocytes, monocytes, and neutrophils levels were observed. However, hemoglobin and hematocrit values for female* O. niloticus* were significantly lower than those for male* O. niloticus*. Organosomatic indices showed that the relative liver, kidney, and spleen weights were significantly higher (*P* < 0.05) in the AFB_1_ supplemented group than in the control group. However, the effect of aflatoxin on organosomatic indices does not depend on sex but rather depends on the dose of aflatoxin in the diet. These results provide useful information for monitoring changes in the health status of male and female* O. niloticus*.

## 1. Introduction

*Oreochromis niloticus* are currently the main cultured fish species in East Africa (accounting for 75%), and they contribute the bulk of fish, yielding approximately 3,400 tons (69.9%) [1]. In* O. niloticus*, diets are predominantly formulated using high levels of grain and plant protein and are at high risk of being contaminated by aflatoxin [2]. Aflatoxin (AF), a potent mycotoxin produced by* Aspergillus flavus* and* Aspergillus parasiticus*, is a top challenge in aquaculture production due to the increasing use of vegetable sources in fish feeds [3]. Aflatoxin contamination in fish causes anemia, hemorrhaging, liver damage, weight reduction, vulnerability to secondary infectious diseases, and a slow decrease in reared fish stock quality, thus representing a significant challenge in aquaculture [4, 5].

Information on various infections of visceral organs, necrosis, inflammation, and the presence of stress factors is generally provided by hematological parameters [6, 7]. These parameters are associated with the blood and blood-forming organs [8]. Blood acts as a pathological reflector of the status of animals exposed to the toxicant and other conditions; animals with good blood composition likely show better performance [9, 10]. The study of the physiological and hematological characteristics of cultured fish species is an essential tool in the advancement of aquaculture systems, especially with respect to the detection of healthy versus infected or stressed fish [11, 12]. In addition, Ronald and Bruce [13] described organosomatic indices as the proportion of organs to body weight; an organ measured in relation to body mass can be directly connected to toxic effects by a chemical to the target organ [14]. The most commonly used organosomatic indices are the gonadosomatic index (GSI), spleen somatic index (RSI), kidney somatic index (KSI), and hepatosomatic index (HSI) [15, 16]. These organosomatic indices may provide more specific information related to the function of the selected organ [15].

The toxic effect of aflatoxin in animals depends on the dose in the feeds and duration of toxin exposure, as well as the sex and age [17–19]. Sex-related differences in aflatoxin tolerance have been reported in pigs, broilers, and rats and these studies reported that males are more susceptible to aflatoxin than their female counterparts [20–22]. Despite the vast number of studies on the effects of aflatoxin on hematology and organosomatic indices of different species of fish, no study investigated whether males and females of* O. niloticus* exhibit differences in hematological responses and organosomatic indices to dietary AFB_1_ contamination. Therefore, this study evaluates sex-related differences in hematological responses and organosomatic indices of* O. niloticus* exposed to an aflatoxin B_1_-contaminated diet.

## 2. Materials and Methods

### 2.1. Experimental Fish and Procedure

A total of 300* O. niloticus* weighing 24.1 ± 0.6 g were obtained from the National Aquaculture Research and Development Centre (NARDTC), Sagana, Kenya. Fish were acclimatized to the trial condition and fed with the control diet for 15 days. A total of 300* O. niloticus* were divided into twelve equal groups of 25 fish. Each treatment was divided into 3 replicates of 25 fish each and kept in cages. Fish were fed to apparent repletion twice a day at 0900 and 1500 hr. To minimize feed wastage, pellets were slowly fed to the fish. The experiment was performed in 12 cages (1 m^3^) placed in three different ponds (40 × 20 m).

During the experiment, the water quality parameters were as follows: temperature 24.2–26.8°C, pH 7.5–7.65, dissolved oxygen 4.6–6.3 mg L^−1^, total alkalinity 146.65–225.95 mg L^−1^, and salinity 0.03–0.05. The trial lasted for 24 weeks. Females and males in each experiment unit were checked monthly to determine the number, weight, length, and any physiological changes. Twenty fish from each cage were sampled randomly after 1 day of food deprivation near the end of the experiment.

### 2.2. Experimental Diets

A pure crystalline powder of AFB_1_ was purchased from the Libios Chemical Company, Rue Edmond Michelet, France. AFB_1_ (5 mg) was dissolved in 100 mL of methanol to form a stock solution containing 0.05 mg AFB_1_ mL^−1^ of methanol. Four experimental diets (Diets 1 to 4) were formulated to contain 0, 20, 200, and 2000 *μ*g AFB_1_ kg^−1^ diets. The control diet was Diet 1, without supplementary chemical AFB_1_.

All diets were processed with the aid of a meat grinder with approximately 32% moisture and pelleted, followed by 4 hr of drying in an air flow oven at 60°C until the moisture content was less than 10%. The dry pellets (5 mm diameter) were stored at −20°C until fed.  Table 1 shows the composition and nutrient content of the diets.

The given toxin levels were finally cross-checked using an Enzyme-Linked Immunosorbent Assay (ELISA) (BioTek Instruments, Inc., VT, USA).

### 2.3. Relative Organ Weight

At the end of the 24-week experiment, the fish were carefully netted to minimize stress, and the fish was measured (total length) with an ichthyometer (±1 cm precision) and weighed. After this, the fish were anesthetized with sodium bicarbonate and sacrificed. The liver, spleen, and kidney were carefully removed and weighed.

The organosomatic indices of the liver, spleen, and kidney were calculated for the twenty fish according to Dogan and Can [23] to obtain the ratios of organ weight to body weight for the fish.

### 2.4. Blood Parameters Analysis

Blood specimens were taken from the caudal vein in heparinized tubes and centrifuged at 3,500 g for 10 min; serum samples were separated and stored at −20°C until analysis. The analyzed serum parameters such as alanine aminotransferase (ALT) were determined using an automatic analyzer (Beckman Coulter DU @ 530, automatic biochemical analyzer, USA).

Hemoglobin (Hb) concentration was evaluated by Sahli's hemometer [24] and hematocrit (Hct) concentration was determined using the microhematocrit method [25]. Red blood cells (RBC) and white blood cells (WBC) were counted using the improved Neubauer hemocytometer [25], and for differential leucocyte counts, freshly prepared blood smears were stained with modified Giemsa's stain [26] and observed under a microscope. The mean corpuscular volume (MCV), mean corpuscular hemoglobin (MCH), and mean corpuscular hemoglobin concentration (MCHC) were calculated as stated by Mahfouz and Sherif [27].

### 2.5. Histopathological Procedure

From each treatment, ten fish were sacrificed to acquire liver samples for histology and tissue analysis. Bouin's solution was used to store samples for the first 24 hr, and then samples were stored in 70% ethanol. Tissue samples were dehydrated in a graded ethanol series and embedded in paraffin before being processed. Tissue blocks were subsequently sectioned and stained with hematoxylin and eosin [28] and then examined under a microscope for common and/or significant lesions and associated histopathological changes of aflatoxicosis [29].

### 2.6. Data Analysis

Data analyses were performed using a two-way ANOVA for the main effects of aflatoxin, sex, and their interaction using the General Linear Model procedure of SPSS (version 16.0). Significant means (*P* < 0.05) were separated using Tukey's Test. Data are presented as the means ± SEM.

## 3. Results

### 3.1. Relative Organ Weight and Physiology Response

Data for internal organs indices of the tested* O. niloticus* are in Table 2. Aflatoxin B_1_ significantly increased the HSI, KSI, and SSI (*P* < 0.05). The HSI, KSI, and SSI in the control group had the lowest values (0.88 g, 0.15 g, and 0.05 g, resp.) compared to Diet 4 (2.97, 0.42, and 0.34 g, resp.). The HSI and KSI significantly increased in Diets 2, 3, and 4 but SSI increased in Diets 3 and 4. The HSI, KSI, and SSI revealed no sex differences in response to aflatoxin B_1_ (Table 2).

Aflatoxin B_1_ also significantly increased the activity of the serum ALT liver enzyme (*P* < 0.05). The control group showed a low activity of the ALT liver enzyme (41.96 UL^−1^) compared to that in Diet 4 (50.69 UL^−1^). Serum ALT revealed no sex differences in response to AFB_1_.

### 3.2. Hematological Characteristics

The inclusion of different aflatoxin levels in the diet significantly (*P* < 0.05) affected RBC in both sexes, with the highest value (2.96) in the control group compared to AFB_1_ supplemented diets (1.98, 1.43, and 1.01 for Diets 2, 3, and 4, resp.) in Table 3.

There were significantly decreased Hct and Hb in both sexes of* O. niloticus* on Diets 2, 3, and 4 compared to controls (Table 3). Hct decreased by 30.00, 24.50, and 20.25% (for Diets 2, 3, and 4, resp.) compared to the control group value (38.50%) in Table 3. However, MCV and MCH of male and female fish were significantly (*P* < 0.05) higher in the AFB_1_ supplemented diet than in the control diet. The highest MCV was recorded in fish fed with Diet 4 (198.7 *μ*m^3^), while the lowest was recorded in the control group (129.8 *μ*m^3^). There was no significant difference in RBC and MCHC between sexes. The statistical comparison between male and female* O. niloticus* revealed a significant difference for Hb (*P* < 0.05), Hct (*P* < 0.05), MCV (*P* < 0.05), and MCH (*P* < 0.05) in Table 3. Female* O. niloticus* fed with Diets 2, 3, and 4 recorded lower Hb (7.52, 4.05, and 3.55 gdL^−1^) than male* O. niloticus* (7.72, 4.27, and 3.67 gdL^−1^).

The total WBC counts of fish of both sexes from Diet 3 were higher than in the control group due to an increase in lymphocytes and neutrophils. Absolute monocyte counts of fish fed with the AFB_1_ supplemented diets were much lower than in the control group (Table 2). There were no significant sex differences (*P* > 0.05) in WBC, lymphocytes, neutrophils, and monocytes among all treatments. The hematocrit values presented a strong positive correlation with the length of the fish (*P* < 0.05, *r* = 0.939). As the aflatoxin dose increases, the fish length significantly decreases (Table 3).

### 3.3. Histopathology

**Figure 1:**
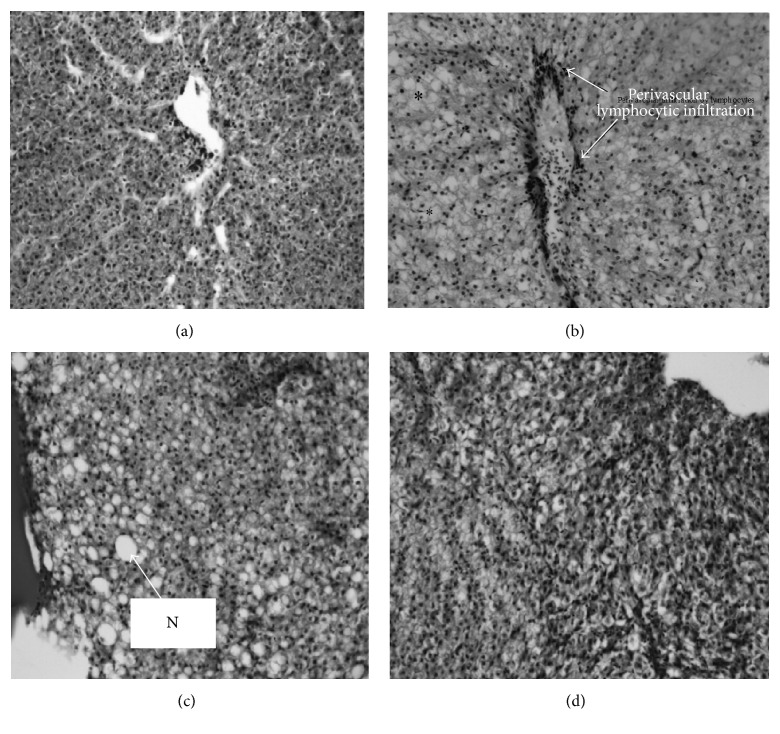
Histology of a liver in* O. niloticus* fed with Diet 1 (a), Diet 2 (b), Diet 3 (c), and Diet 4 (d) for 24 weeks. H&E; bar = 400 *μ*m. ^*∗*^N: fatty necrosis.

Histopathological examinations revealed that fish liver from the control treatment had normal hepatocytes arranged in trabecula forming a centripetal sinusoid (Figure 1(a)). However, the trabecular arrangement of hepatocytes was less apparent in fish fed with Diet 2, with the presence of multifocal lymphocytic infiltration including the perivascular region (Figure 1(b)). Hepatocytes showed hemorrhagic and fatty necrosis, as evidenced by vacuolization and hemorrhage (Figure 1(c)). Some cells were granular in appearance, with enlarged nuclei indicative of necrosis (Figures 1(c) and 1(d)). The severity of liver pathology varied with low and high doses of aflatoxin B_1_. In the liver with low-dose treatments, there were early signs of necrosis, as indicated by cell vacuolization with normal nuclei (Figure 1(c)). In the livers with a high dose, there was late-stage necrosis indicated by enlarged cell nuclei, karyolysis, and cytoplasmic disintegration (Figure 1(d)).

## 4. Discussion

In this study, we evaluated the sex-related differences in the hematological responses and organosomatic indices of* O. niloticus* exposed to aflatoxin B_1_-contaminated diet and found differences among the sexes.

In the present study, RBC, Hct, Hb, and MCHC levels decreased significantly (*P* < 0.05) in the high AFB_1_ exposed group compared with the control group. In the AFB_1_ exposed group,* O. niloticus* suffered from anemic conditions as revealed by a marked decrease in RBC and Hb. The diminished RBC, Hct, and hemoglobin may be due to factors such as inhibited protein synthesis, erythropoiesis, hemosynthesis, and osmoregulatory dysfunction owing to repressed activities of several enzymes involved in heme biosynthesis [27] and hematopoietic cellular defects of aflatoxin [30]. Our results corroborate the studies of Mahfouz and Sherif [27] and Jantrarotai and Lovell [31], who observed a decrease in hemoglobin concentration and erythrocyte counts in* O. niloticus* and channel catfish exposed to AFB_1_ diets.

The present study also assessed whether male or female* O. niloticus* exhibit differences in their hematological response when exposed to AFB_1_ diet. Because of the wide use of male monosex tilapia, sex-based differences in the hematological parameters should be determined if male* O. niloticus* are more resistant when exposed to AFB_1_ diet. According to our results, no sex-related differences in RBC and MCHC levels were observed. However, hemoglobin and hematocrit values for males were significantly higher than for female* O. niloticus.* We suspect that the differences may not be because of the toxicity effect of AFB_1_ but due to the higher metabolic rate of males compared to females or a sex-differential oxygen requirement and this requires further investigation.

A higher MCV is an indicator of macrocytic anemia, while a reduced MCHC is indicative of anemic conditions due to iron deficiency [32, 33]. Similar to our results, a higher MCV and lower MCHC were observed in* O. niloticus* exposed to AFB_1_ compared to the control group. Our results were also supported by the findings of Ewuola et al. [34] and Weaver et al. [35] who found an increase in MCV and a decrease in MCHC of goat and pig fed with an AFB_1_-contaminated diet. Gabriel et al. [36] reported that there is a relationship between blood hematocrit and body length: longer fish have higher hematocrit in* C. carpio*. This implies that rapid increases in mean body weight and length and blood volume are accompanied by adequate erythropoiesis [37]. This is in agreement with our findings that* O. niloticus* exposed to AFB_1_ were shorter and hence had lower Hct.

In the present study, we found that WBC, lymphocytes, monocytes, and neutrophils were influenced by the AFB_1_ dose and not the sex of* O. niloticus*. Significantly, greater WBC and lymphocytes were observed in aflatoxin exposed groups, which may be attributed to the immunosuppressive effect of aflatoxin on fish. These results are in contrast with those of Mahfouz, [38] who reported that WBC, lymphocytes, and neutrophils were decreased in* O. niloticus* fed with an AFB_1_ supplemented diet. However, our results are in accordance with those of Quist et al. [39] and Jantrarotai and Lovell [31], who observed increased WBC and lymphocytes in wild turkey pouts and channel catfish fed with an AFB_1_ supplemented diet. WBC increased with infection, first cooperating to recognize the pathogen as an invader and destroy it. There was an increase in WBC during infection and allergic responses in the host [40]. Therefore, aflatoxin might enhance the invasion of pathogens in tested animals, giving way to opportunistic infections and a subsequent increase in WBC [40].

In this study, the hepatosomatic index (HSI), kidney somatic index (KSI), and spleen somatic index (SSI) disclosed no sex differences in response to aflatoxin B_1_. These results are in agreement with the previous study of Bryden et al. [20], who found that the hepatosomatic index of male and female chicken fed with AFB_1_ does not depend on sex. The HSI, KSI, and SSI of* O. niloticus* (both sexes) exposed to AFB_1_ supplemented diets were found to be significantly higher than in the control group. These findings are in concurrence with past studies where AFB_1_ resulted in an increase in HSI and KSI [41–45]. Additionally, serum ALT shows no sex differences in response to aflatoxin B_1_. The levels of ALT were found to be significantly higher in AFB_1_ exposed fish. Similarly, previous reports show that AFB_1_ resulted in an increase in HSI and increased activities of plasma ALT [41–43]. Elevation of ALT activity in the serum is an indication of cellular damage and liver tissue necrosis following the exposure of* O. niloticus* to AFB_1_ [46]. This is confirmed by our histological results, in which fatty necrosis, hemorrhagic necrosis, and enlarged cell nuclei with vacuolized cytoplasm were identified as the most prevalent histological characteristics in fish fed with AFB_1_ diet. These observations are comparable with previous histopathological findings by Zychowski et al. [47] and Baptista et al. [48] who reported hepatocellular lipid deposition, hemorrhagic necrosis, and enlarged cell nuclei with vacuolized cytoplasm in the livers of rats and* O. niloticus* fed with an aflatoxin diet.

## 5. Conclusion

The hematological and organosomatic indices measured in the present study are useful for monitoring the effects of aflatoxin on fish. This work concluded that aflatoxin is highly toxic to both male and female* O. niloticus*. Elevation of ALT activity in the serum and HSI for both male and female* O. niloticus* appears to reflect histopathological alterations in the liver of the AFB_1_ exposed fish. Aflatoxin increases the WBC and lymphocytes and reduces RBC, Hct, and Hb of both male and female* O. niloticus*. However, hemoglobin and hematocrit values were lower in female* O. niloticus* than in male* O. niloticus*. Further studies to determine the mechanism of action of AFB_1_ in female and male* O. Niloticus* hematological system are recommended.

## Figures and Tables

**Table 1:** Ingredients and nutrient composition of the basal diet of *O. niloticus*.

Ingredients^*∗*^	Content (g/100 g diet)
Soybean meal	30
Shrimp meal	17.9
Wheat bran	5
Sunflower seedcake	40.57
Sunflower oil	2
Cassava flour	1.5
Vitamin premix^1^	1.06
Starch	1.97
*Chemical analysis (% or kJ g* ^−*1*^ * in dry matter)*	
Crude protein	30.83
Ether extract	5.93
Ash	5.93
NFE	51.56
GE (Kcal/g)	441.89

NFE: nitrogen-free extract; GE: gross energy. ^1^Vitamin premix (mg kg^−1^ diet): vitamin A, 18 MIU; vitamin D3, 4 MIU; vitamin E, 6.5 g; vitamin B2, 3.5 g; vitamin K3, 2 g; nicotinic acid, 17 g; pantothenic acid, 7 g; folic acid, 0.4 g; vitamin B1, 1.5 g; vitamin B6, 2.5 g; vitamin C, 12 g; magnesium, 6 g; potassium, 7.5 g; sodium, 20 g; citric acid, 18 g. ^*∗*^Experimental doses of AFB_1_ were 0, 20, 200, and 2,000 *μ*g kg^−1^; measured levels were 0.68, 22.5, 235.4, and 2246.5 *μ*g AFB_1_ kg^−1^, respectively.

**Table 2:** Mean ± SE of relative organ weights (g organ per 100 g body weight), white blood cells, differential counts, and liver enzymes (e.g., alanine aminotransferase (ALT)) of male and female *O. niloticus* exposed to different levels of AFB_1_ for 24 weeks.

Diet^*∗*^	Sex^†^	HSI	KSI	SSI	WBC 10^3^	Monocytes%	Neutrophils%	Lymphocytes%	ALT (UL^−1^)
1	F	0.88 ± 0.03^a^	0.15 ± 0.002^a^	0.05 ± 0.001^a^	19.30 ± 0.28^a^	3.56 ± 0.003^a^	0.37 ± 0.001^a^	10.82 ± 0.2^a^	41.96 ± 0.08^a^
2		1.42 ± 0.02^b^	0.24 ± 0.001^b^	0.07 ± 0.001^a^	20.29 ± 0.24^a^	3.05 ± 0.005^b^	0.26 ± 0.008^b^	16.50 ± 0.01^b^	42.56 ± 0.06^a^
3		1.94 ± 0.05^c^	0.33 ± 0.003^c^	0.11 ± 0.002^b^	28.26 ± 0.85^b^	0.52 ± 0.004^c^	2.01 ± 0.01^c^	29.21 ± 0.18^c^	46.82 ± 0.05^b^
4		2.97 ± 0.04^d^	0.42 ± 0.005^d^	0.34 ± 0.12^c^	37.97 ± 0.08^c^	0.37 ± 0.003^d^	3.97 ± 0.005^d^	30.75 ± 0.03^cd^	50.50 ± 0.08^c^

1	M	0.84 ± 0.03^a^	0.15 ± 0.002^a^	0.05 ± 0.001^a^	19.45 ± 0.29^a^	3.58 ± 0.03^a^	0.38 ± 0.001^a^	10.95 ± 0.02^a^	41.99 ± 0.06^a^
2		1.40 ± 0.02^b^	0.24 ± 0.001^b^	0.07 ± 0.001^a^	20.47 ± 0.25^a^	3.06 ± 0.05^b^	0.28 ± 0.006^b^	16.70 ± 0.01^b^	42.60 ± 0.08^a^
3		1.89 ± 0.05^c^	0.34 ± 0.003^c^	0.11 ± 0.02^b^	28.45 ± 0.85^b^	0.54 ± 0.03^c^	2.02 ± 0.01^c^	29.36 ± 0.10^c^	46.84 ± 0.08^b^
4		2.94 ± 0.04^d^	0.42 ± 0.004^d^	0.33 ± 0.13^c^	38.16 ± 0.08^c^	0.38 ± 0.03^d^	3.98 ± 0.004^d^	30.83 ± 0.06^d^	50.69 ± 0.05^c^

*ANOVA*									
Aflatoxin^*∗*^ sex		0.97	1	1	1.00	0.99	0.99	0.98	0.68
Sex		0.10	0.62	0.98	0.62	0.19	0.33	0.18	0.25
Aflatoxin (dose)		<0.05	<0.05	<0.05	<0.05	<0.05	<0.05	<0.05	<0.05

^†^M: male* O. niloticus*; F: female *O. niloticus *with *n* = 10 (10 individual fish per treatment (10 males and 10 females) were measured); ^*∗*^diet 1: 0 *μ*g kg^−1^ (control); diet 2: 20 *μ*g kg^−1^; diet 3: 200 *μ*g kg^−1^; diet 4: 2000 *μ*g kg^−1^. ^*∗*^Means followed by similar letters do not differ significantly (*P* < 0.05).

**Table 3:** Mean ± SE of hematological parameters of male and female *O. niloticus *exposed to different levels of AFB_1_ for 24 weeks.

Diet^*∗*^	Sex^†^	Length (cm)	RBC ×10^6^	Hb (g dL^−1^)	HCT%	MCV (*μ*m^3^)	MCH (pg)	MCHC%
1	F	21.67 ± 0.76^a^	2.96 ± 0.01^a^	8.02 ± 0.07^a^	38.50 ± 0.6^a^	129.8 ± 1.75^a^	27.06 ± 0.15^a^	25.13 ± 0.62^b^
2		20.27 ± 0.43^b^	1.98 ± 0.80^b^	7.52 ± 0.04^b^	30.00 ± 0.7^b^	151.1 ± 3.50^b^	37.89 ± 0.25^b^	20.85 ± 0.17^a^
3		18.92 ± 0.13^c^	1.43 ± 0.02^c^	4.05 ± 0.06^c^	24.50 ± 0.5^c^	170.4 ± 3.20^c^	28.16 ± 0.32^c^	16.56 ± 0.49^c^
4		17.05 ± 0.34^d^	1.01 ± 0.67^d^	3.55 ± 0.02^d^	20.25 ± 0.4^d^	198.7 ± 4.80^d^	34.83 ± 0.22^d^	17.56 ± 0.39^c^

1	M	22.22 ± 0.86^a^	2.97 ± 0.01^a^	8.15 ± 0.09^a^	40.50 ± 0.65^a^	136.5 ± 1.73^a^	27.46 ± 0.23^a^	20.13 ± 0.12^a^
2		20.12 ± 0.45^b^	1.99 ± 0.85^b^	7.72 ± 0.05^b^	32.00 ± 0.70^b^	161.0 ± 3.52^b^	38.86 ± 0.25^b^	24.18 ± 0.56^b^
3		18.37 ± 0.05^c^	1.44 ± 0.02^c^	4.27 ± 0.04^c^	26.50 ± 0.50^c^	184.1 ± 3.30^c^	29.69 ± 0.29^c^	16.15 ± 0.39^c^
4		17.22 ± 0.38^d^	1.02 ± 0.67^d^	3.67 ± 0.05^d^	21.50 ± 0.50^d^	210.8 ± 4.40^d^	36.02 ± 0.27^d^	17.11 ± 0.34^c^

*ANOVA*								
Aflatoxin^*∗*^ sex		0.18	1.00	0.77	0.89	0.76	0.19	0.980
Sex		0.97	0.79	<0.05	<0.05	<0.05	<0.05	>0.05
Aflatoxin (dose)		<0.05	<0.05	<0.05	<0.05	<0.05	<0.05	<0.05

^†^M: male* O. niloticus*; F: female *O. niloticus *with *n* = 10 (10 individual fish per treatment (10 males and 10 females) were measured); ^*∗*^diet 1: 0 *μ*g kg^−1^ (control); diet 2: 20 *μ*g kg^−1^; diet 3: 200 *μ*g kg^−1^; diet 4: 2000 *μ*g kg^−1^. ^*∗*^Means followed by similar letters do not differ significantly (*P* < 0.05).
